# Genetic characterization of the human relapsing fever spirochete *Borrelia miyamotoi* in vectors and animal reservoirs of Lyme disease spirochetes in France

**DOI:** 10.1186/1756-3305-7-233

**Published:** 2014-05-20

**Authors:** Jean-François Cosson, Lorraine Michelet, Julien Chotte, Evelyne Le Naour, Martine Cote, Elodie Devillers, Marie-Lazarine Poulle, Dominique Huet, Maxime Galan, Julia Geller, Sara Moutailler, Muriel Vayssier-Taussat

**Affiliations:** 1 INRA, UMR1062, CBGP, F-34988 Montferrier-sur-Lez, France; 2 INRA, USC Bipar, Anses, 23 Avenue du Général de Gaulle, Maisons-Alfort, France; 3 URCA, SFR Cap Santé, PROTAL EA 3800, Reims, France; 4 URCA, CERFE, 08240 Boult-aux-Bois, France; 5 National Institute for Health Development, Tallinn, Estonia

**Keywords:** Co-infection, Lyme disease, Bank voles, Ticks

## Abstract

**Background:**

In France as elsewhere in Europe the most prevalent TBD in humans is Lyme borreliosis, caused by different bacterial species belonging to *Borrelia burgdorferi* sensu lato complex and transmitted by the most important tick species in France, *Ixodes ricinus*. However, the diagnosis of Lyme disease is not always confirmed and unexplained syndromes occurring after tick bites have become an important issue. Recently, *B. miyamotoi* belonging to the relapsing fever group and transmitted by the same *Ixodes* species has been involved in human disease in Russia, the USA and the Netherlands. In the present study, we investigate the presence of *B. miyamotoi* along with other Lyme Borreliosis spirochetes, in ticks and possible animal reservoirs collected in France.

**Methods:**

We analyzed 268 ticks (*Ixodes ricinus*) and 72 bank voles (*Myodes glareolus*) collected and trapped in France for the presence of DNA from *B. miyamotoi* as well as from Lyme spirochetes using q-PCR and specific primers and probes. We then compared the French genotypes with those found in other European countries.

**Results:**

We found that 3% of ticks and 5.55% of bank voles were found infected by the same *B. miyamotoi* genotype, while co-infection with other Lyme spirochetes (*B. garinii*) was identified in 12% of *B. miyamotoi* infected ticks. Sequencing showed that ticks and rodents carried the same genotype as those recently characterized in a sick person in the Netherlands.

**Conclusions:**

The genotype of *B. miyamotoi* circulating in ticks and bank voles in France is identical to those already described in ticks from Western Europe and to the genotype isolated from a sick person in The Netherlands. This results suggests that even though no human cases have been reported in France, surveillance has to be improved. Moreover, we showed that ticks could simultaneously carry *B. miyamotoi* and Lyme disease spirochetes, increasing the problem of co-infection in humans.

## Background

Human pathogenic *Borrelia* species are comprised of two main groups of spirochetes: the Lyme borreliosis group which cause human Lyme diseases, are widespread throughout Europe, Asia and North America and are transmitted between vertebrates by hard ticks (genus *Ixodes*) [1]; and the group causing severe relapsing fever in humans and which are transmitted by both, soft and hard ticks [2]. *Borrelia miyamotoi,* belonging to the relapsing fever group, is transmitted by the same *Ixodes* species that also transmits *Borrelia* species from the Lyme Borreliosis group. *B. miyamotoi* was isolated for the first time in Japan in 1995 from *I. persulcatus* ticks as well as from the blood of *Apodemus argentus* mice [3,4], and over recent decades, it has also been detected in *Ixodes* ticks in Europe, Russia and the USA [5-7]. The first human cases of *B. miyamotoi* infection were reported in Russia in 2011 [8], and more recently, human infections have been described in the USA and the Netherlands [9-12].

In Europe, Lyme borreliosis is the most common tick-borne disease and is caused by different bacterial species belonging to the *Borrelia burgdorferi* sensu lato complex. They are transmitted by the tick species which has the most impact on European public health, *Ixodes ricinus*, and which are hosted by many animal species, like rodents, deer, birds and lizards*.* In France, over 10,000 new cases of Lyme borreliosis are estimated to occur each year. When there has been a history of tick bites, Lyme disease is commonly assumed, but in some cases there is an absence of diagnostic confirmation. Besides Lyme borreliosis typical cases, diagnosed in its first stage by the presence of an erythema migrans following tick bites and in its later phases by serological tests, a number of patients complain of polymorphic and unspecific clinical symptoms (asthenia, fever, myalgia, etc…), for which the diagnosis is not straightforward [13]. In recent years, unexplained syndromes occurring after tick bite has thus become a very important issue, grown considerably with alternate interpretations of the Lyme disease serology, which has led to considerable unrest between formal institutions of infectious disease and patient associations. The proportion of patients bitten by ticks with unexplained syndromes compared with patients who contracted Lyme disease is difficult to know precisely although it is estimated that 50% of fever after tick bites are of unknown etiological origin [14]. Unexplained syndromes, which have occurred following tick bites have thus become a very important issue in recent years.

In order to better characterize those tick-borne pathogens that are likely to be involved in unexplained tick-borne diseases, we recently generated a global picture of their tick-borne bacteria by analyzing whole transcriptomes of *I. ricinus* ticks. Surprisingly, we identified the unanticipated *B. miyamotoi* in *I. ricinus* collected in France [15]. In the present study, our aim was to investigate the presence and prevalence of *B. miyamotoi*, along with Lyme borreliosis spirochetes, in both their vectors, i.e., *I. ricinus*, and their possible animal reservoirs (bank voles, *Myodes glareolus*). We have established that *I. ricinus* and bank voles are infected with *B. miyamotoi* along with Lyme disease spirochetes, that the same *B. miyamotoi* genotype circulate in both *I. ricinus* as well as in the bank voles and that this genotype is perfectly identical to those described in Western Europe, including those infecting humans [12].

## Methods

### Ticks and rodents

Ticks (268 questing *Ixodes ricinus* female ticks) and bank voles (72 *Myodes glareolus*) were collected and trapped in the French Ardennes, an endemic region for rodent-borne hantaviruses, along a transect line of about 80 km in the course of a study of the epidemiology of *Puumala* hantavirus [16]. Along this transect, we sampled six sites in forested areas and four sites in fragmented habitats (i.e. hedge network). Animals were killed by cervical dislocation. They were weighed, sexed and dissected. All collected ticks were washed and crushed as previously described [17].

### DNA extraction

Voles genomic DNA was extracted from a piece of spleen using the DNeasy kit (Qiagen) according to manufacturer’s instructions with a final elution volume of 100 μL. Tick genomic DNA was extracted using the Wizard genomic DNA purification kit (Promega) according to manufacturer’s instructions with a final elution volume of 50 μL.

### Specific Real-Time PCR for detection and prevalence of *Borrelia* spp. in ticks and bank voles

Quantitative real-time PCR was performed to determine the prevalence of *B. miyamotoi, B. afzelii*, *B. burgdorferi* sensu stricto, *B. garinii*, *B. valaisiana* and *B. spielmanii* using primers and probes described in [15]. A DNA pre-amplification step was performed in a final volume of 5 μl containing 2.5 μl TaqMan PreAmp Master Mix (2X), 1.2 μl of the pooled primer mix (0.2X) and 1.3 μl of ticks or rodent DNA, with one cycle at 95°C for 10 min, 14 cycles at 95°C for 15 sec and 4 min at 60°C. After the pre-amplification PCR, products were diluted 1:20 and stored at -20°C prior to use in qPCR. All fluorogenic probes were synthesized with a 6-carboxy-fluorescein (FAM) reporter molecule attached to the 5’ end and a Black Hole Quencher 1 (BHQ1) attached to the 3’ end. Real-time Taqman PCR assays were performed in a final volume of 12 μl using the LightCycler® 480 Probe Master mix (Roche Applied Science, Germany) at a 1X final concentration, with primers and probes at 200 nM and pre-amplified DNA. Thermal cycling conditions were as follows: 95°C for 5 min, 45 cycles at 95°C for 10s and 60°C for 15 s and then 40°C for 10 s.

### Phylogenetic analysis of *B. miyamotoi* genotypes

Three genomic regions, including partial sequences of *16S rRNA* (1256 bp), *glpQ* (379 bp) and *p66* (532 bp) genes were amplified and sequenced as previously described [18] from *B. miyamotoi* positive-testing tick and rodent DNA extracts. Sequence alignment and phylogenetic analysis was performed using SeaView version 4.4.2 as previously described [19].

### Ethical approval

Animals have been treated in accordance with the guidelines of the European Union legislation (Directive 86/609/EEC). The CBGP laboratory has received the approval (no. B 34-169-1) from the regional Head of Veterinary Service (Hérault, France), for the sampling and killing of rodents and the harvesting of their tissues. Dr Cosson has personally received the agreement “*certificat d'autorisation d'expérimenter sur animaux vivants*” (i.e. “*certificate of authorization to experiment on live animals*”) (no. C34-105) by the French administration.

## Results and discussion

Tick and bank vole DNA samples were assessed for the presence of *B. miyamotoi* via quantitative real-time PCR using primers and probes described in [15]. The prevalence of *B. miyamotoi* in ticks and bank voles was 3.0% (8/267 ticks) and 5.55% (4/72) respectively. The prevalence of *B. miyamotoi* in ticks was similar to that reported for *I. ricinus* collected from other European countries, which ranged from 0.4% in Estonia to 3.5% in Germany [5,18]. To our knowledge, this study provides the first estimation of *B. miyamotoi* DNA prevalence in bank voles. Interestingly, ticks and rodents positive for *B. miyamotoi* were trapped from very diverse terrains within our study site, including large expanses of forest (near Les Woiries, 49.903°N, 4.766°E; and Renwez, 49.859°N, 4.611°E), forest patches (near La Cassine, 49.575°N, 4.794°E; and Elan 49.653°N, 4.766°E) and hedge networks bordering grasslands (near Sauville, 49.545°N, 4.801°E; Boult-aux-Bois 49.413°N, 4.920°E), also with close proximity to human dwellings in some cases.

In addition to *B. miyamotoi*, we also detected Lyme spirochetes *B. afzelii* in 7.11% of ticks and 4.16% of bank voles, and we also found *B. burgdorferi* sensu stricto, *B. garinii*, *B. valaisiana* and *B. spielmanii* in 1.87%, 9.74%, 5.24% and 1.12% of ticks respectively. Co-infection between *B. miyamotoi* and *B. garinii*, was noted in one tick (corresponding to 12.00% [1/8] of *B. miyamotoi*-infected ticks).

*B. miyamotoi* has previously been isolated from the blood of different rodent species such as the small Japanese field mouse (*Apodemus argentus*) from Japan [3], and the white-footed mouse (*Peromyscus leucopus*) from the USA [6], all of these species are likely potential *B. miyamotoi* host reservoirs. Our results support the importance of different rodent species in the circulation of *B. miyamotoi*, by adding the bank vole (*Myodes glareolus*) in the list of potential *B. miyamotoi* reservoirs in Europe.

In order to better characterize *B. miyamotoi* genotypes, three genomic regions, including partial sequences of *16S rRNA* (1256 bp), *glpQ* (379 bp) and *p66* (532 bp) genes were amplified and sequenced as previously described [18] from positive-testing tick and rodent DNA extracts. All three genes were amplified in all *B. miyamotoi* positive tick DNA samples, but the *16S RNA* gene was amplified in four *B. miyamotoi* positive bank vole DNA samples, *glpQ* in only three, and the *p66* gene was not amplified at all in bank vole DNA samples.

**Figure 1:**
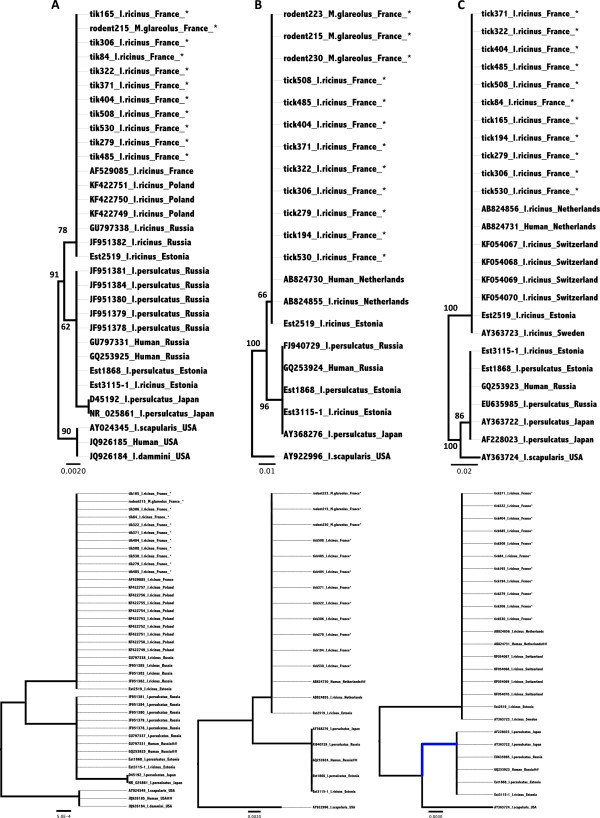
**Phylogenetic trees of B. miyamotoi genotypes based on the partial sequences of A) *16S rRNA*(GenBank accession numbers of the French data from KJ412189 to KJ412199), B) *glpQ *(GenBank accession numbers the French data from KJ425352 to KJ425363) and C) *p66 *genes (GenBank accession numbers the French data from KJ425364 to KJ425374).** Phylogenetic analysis for each of the three genes was performed via the neighbor-joining method and a Kimura 2-parameter distance. Bootstrap analysis was performed on 1,000 replicates. Trees were rooted with *Borrelia turicatae* and *Borrelia ionestari* (not shown). Numbers beside branches indicate bootstrap values. Abbreviations: I. for *Ixodes*, and M. for *Myodes*. Scale bars indicate Jukes-Cantor evolutionary distances. Samples sequenced in the present study are marked with _*.

Nucleotide sequence similarity analysis of the three genomic regions (two for the bank vole samples) showed that all *B. miyamotoi* tick sequences were 100% identical to those amplified from bank voles (Figure 1), suggesting that the same genotype circulates between *I. ricinus* and bank voles. Phylogenetic analyses of these three genes were internally consistent with each other and gave strong support to the proposed genetic structuring of *B. miyamotoi* into three distinct geographic clades of Western Palearctic, Eastern Palearctic and Nearctic respectively (Figure 1). French sequences clustered to those amplified from *I. ricinus* in Switzerland, the Netherlands, Sweden, Estonia, Poland and the European part of Russia. They also clustered with the strain recently described from the patient in the Netherlands [12]. All of these sequences were clearly separated from a second group, which includes sequences from *I. ricinus, I. persulcatus* and human blood from Estonia, Russia and Japan. A third group includes sequences generated from ticks and human tissues from the USA. Finally, it is relevant to note that all three of these *B. miyamotoi* genetic groups include strains that are pathogenic for humans.

## Conclusions

We have established that *B. miyamotoi* is present in *I. ricinus* as well as in the bank vole *Myodes glareolus*, one of the most important sources of blood meals for *I. ricinus* larvae. The genotypes of all *B. miyamotoi* detected in French ticks and rodents were perfectly identical to genotypes already described in ticks from Western Europe, and to the genotype recently isolated from a sick person in the Netherlands. Thus these findings have important implications for public health especially considering that *B. miyamotoi*-positive ticks and rodents were collected from different terrains, which were sometimes in close proximity to human dwellings. Up until now, no human cases have been reported in France but our data combined with the recent case of human infection described in the Netherlands suggest that surveillance needs to be improved. Symptoms caused by *B. miyamotoi* could easily be confused with symptoms caused by other pathogens, which are better known by practitioners in our study area, such as Puumala hantavirus or Lyme spirochetes.

In addition, our data suggest that, in the studied area, ticks could simultaneously transmit *B. miyamotoi* and Lyme disease spirochetes (*B. garinii* in our case) to humans. This raises the problem of co-infection in humans, a poorly studied issue but with strong potential implications and relevance for public health.

## Competing interests

The authors declare that they have no competing interests.

## Authors’ contributions

JFC and MVT conducted the study, analyzed data, and drafted the manuscript, LM, JC, ED, DH carried out DNA extraction, PCR and RT-PCR. ELN, MC, MLP and MG assisted with data collection, JG assisted with designing the study. All authors read and approved the final manuscript.
